# Supplementary material for: Circulation of Fluorescently Labelled Phage in a Murine Model
Source: Viruses. 2021 Feb 14;13(2):297. doi: 10.3390/v13020297 (PMC7917791; doi:10.3390/v13020297)
Supplement: Supplementary file 1 [file viruses-13-00297-s001.pdf]

Supplementary Materials

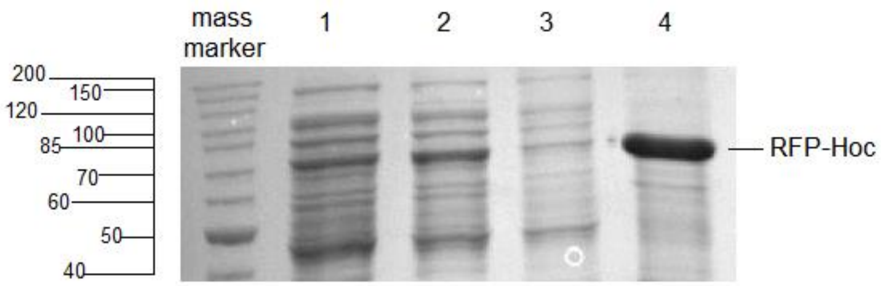

Figure S1. Expression of recombinant RFP-Hoc fusion in *E. coli*. Expression plasmids containing *RFP-hoc* fusion were tested for their effectiveness in production of RFP-Hoc proteins as showed by SDS-PAGE. 1- soluble fraction of the culture before induction (control). 2- soluble fraction of the culture after induction (expression). 3- insoluble fraction of the culture before induction (control). 4- insoluble fraction of the culture after induction (expression).

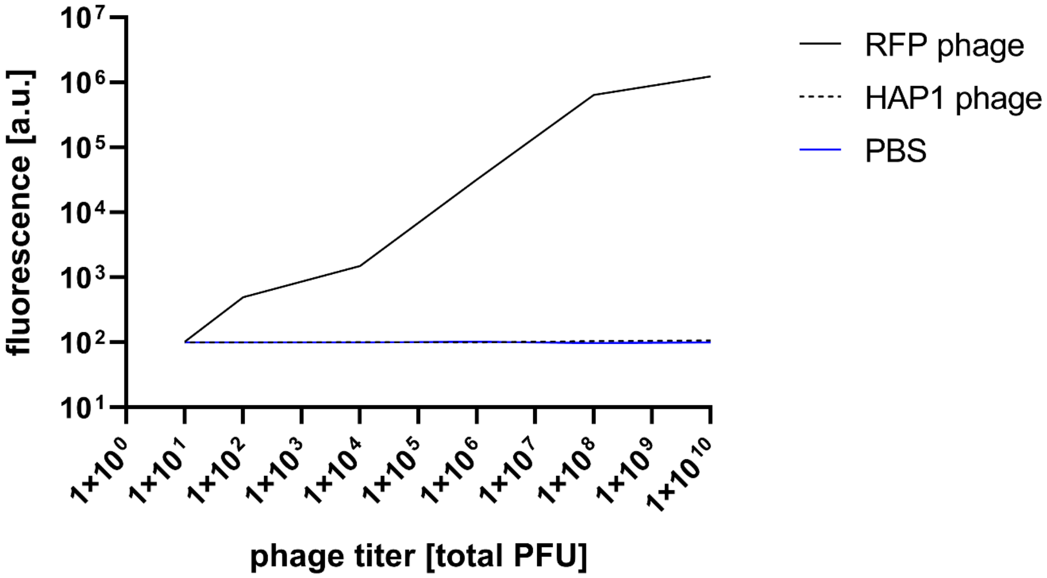

Figure S2. Phage fluorescence of RFP phage (RFP.Hoc.HAP1 - phage labelled with RFP) and HAP1 phage (control phage - non-labelled) in correlation to the phage titer. The fluorescence of pure PBS was also measured (PBS control, blue line).
